# CRISPR-GRANT: a cross-platform graphical analysis tool for high-throughput CRISPR-based genome editing evaluation

**DOI:** 10.1186/s12859-023-05333-w

**Published:** 2023-05-30

**Authors:** Huancheng Fu, Ce Shan, Fanchen Kang, Ling Yu, Zhonghan Li, Yike Yin

**Affiliations:** 1 Center for Growth Metabolism and Aging, Key Laboratory of Bio-Resource and Eco-Environment of Ministry of Education, College of Life Sciences, Sichuan University, Chengdu, China; 2 State Key Laboratory of Biotherapy and Cancer Center, West China Hospital, Sichuan University, Chengdu, China; 3 National Engineering Laboratory for Oral Regenerative Medicine, West China Hospital of Stomatology, Sichuan University, Chengdu, China

**Keywords:** CRISPR, Genome editing, Indel analysis, GUI software, NGS data processing

## Abstract

**Backgroud:**

CRISPR/Cas is an efficient genome editing system that has been widely used for functional genetic studies and exhibits high potential in biomedical translational applications. Indel analysis has thus become one of the most common practices in the lab to evaluate DNA editing events generated by CRISPR/Cas. Several indel analysis tools have been reported, however, it is often required that users have certain bioinformatics training and basic command-line processing capability.

**Results:**

Here, we developed CRISPR-GRANT, a stand-alone graphical CRISPR indel analysis tool, which could be easily installed for multi-platforms, including Linux, Windows, and macOS. CRISPR-GRANT offered a straightforward GUI by simple click-and-run for genome editing analysis of single or pooled amplicons and one-step analysis for whole-genome sequencing without the need of data pre-processing, making it ideal for novice lab scientists. Moreover, it also exhibited shorter run-time compared with tools currently available.

**Conclusion:**

Therefore, CRISPR-GRANT is a valuable addition to the current CRISPR toolkits that significantly lower the barrier for wet-lab researchers to conduct indel analysis from large NGS datasets. CRISPR-GRANT binaries are freely available for Linux (above Ubuntu 16.04), macOS (above High Sierra 10.13) and Windows (above Windows 7) at https://github.com/fuhuancheng/CRISPR-GRANT. CRISPR-GRANT source code is licensed under the GPLv3 license and free to download and use.

## Backgroud

CRISPR (clustered regularly interspaced short palindromic repeats) is a genome-editing technology derived from type II bacterial adaptive immune system, among which CRISPR/Cas9 and CRISPR/Cpf1 are the most widely used [1]. When guided by a short gRNA transcript, CRISPR-Cas could be easily targeted to virtually any genomic loci and generate double-strand DNA breaks by recruiting cellular DNA repair machinery through either non-homologous end joining (NHEJ) or homology-directed repair (HDR), generating DNA insertion or deletion mutations (indels) [2]. Since its discovery, CRISPR has been widely used to understand basic biological processes and has been developed as a potential game-changer for therapeutic applications [3]. However, the off-target effect is still one of the major concerns for CRISPR-mediated genome editing experiments [2], and quantitative analysis of targeted/off-target indels has thus become a standard practice in the lab. Especially with the application of next-generation sequencing (NGS) and routine generation of large-scale datasets [4], systematic analysis of genome edits has become highly dependent on efficient bioinformatics tools.

We reasoned that an ideal bioinformatic program for analyzing CRISPR-mediated genome editing would feature: (1) user-friendly design with graphic user interface (GUI) to guide potential users throughout the process; (2) easy installation in support of cross-platform usage; (3) all-in-one solution to enable both single and multiple amplicon analysis and detection of base-editor mediated single nucleotide changes; (4) locally deployed to avoid uploading of sensitive data or large NGS datasets; (5) highly efficient and could finish whole-genome analysis within a reasonable time frame. To systematically analyze genome edits, currently several tools have been developed, including CRISPResso/CRISPResso2 [5], Cas-analyzer [6], CRISPR-DAV [7], CRIS.py [8], and a few others (Table 1), which could accurately analyze certain kinds of genome editing events, but each has its limitations. They were categorized mainly into two types. One type was locally used, such as CRISPResso, CRIPSResso2 and CRISPR-DAV. These tools often came with command-line based usage, requiring the users had some bio-informatics experiences. Additionally, these command tools were often implemented for Unix-like operating systems, such as Linux, Unix or macOS, not compatible for Windows systems. The other type was web services, such as Cas-analyzer, which had web GUI for online use, convenient for common users. CRISPResso2 also provided web services for amplicon analysis. However, CRISPResso2 only made analysis for amplicon pools or whole-genome available in command-line utilities. Nevertheless, web services could not be used offline, not suitable for NGS data, which was very large, often at the size of megabytes (MB) or even gigabytes (GB). Furthermore, computers in many laboratory holding sensitive data, therefore, usually off the internet, could also not meet the requirement. This would make it difficult for common researchers to use NGS data for CRISPR indel analysis, for they either should have some expertise in bio-informatics or would bear to upload their data to web servers. In addition, among the tools, CRISPResso2, a successor of CRISPResso, was the only one still in heavy development and updating, others, on the contrary, either stopped maintaining or not available for download and use. However, CRISPResso2 was developed in Python2, which had been end of life at April 2020. Therefore, there would be difficulties for users to download and install CRISPResso2 ever since. Furthermore, the amplicon, pooled amplicons and whole-genome analysis command-line tools also had very different interface in CRISPResso2, which might not be friendly for common users. Particularly, most of these tools rely on command line-based usage to analyze target datasets and thus are very difficult for common users such as traditional experimental biologists in the lab. In summary, although several indel analysis tools have been developed, they either require users to have certain bio-informatics training and basic command-line processing capability or need an internet connection during analysis. Therefore, developing a CRISPR indel analysis tool with intuitive GUI, easy installation, and offline cross-platform support is highly desired.

**Table 1:** A list of indel analysis tools which were previously reported

Tools	Platform(s)	Interface	References
CRISPR-GA	NA	NA	Guell et al. [20]
BATCH-GE	Linux	Command line	Boel et al. [21]
CRISPResso	Linux, Docker	Command line	Pinello et al. [22]
CRISPR-DAV	Linux, macOS	Command line	Wang et al. [7]
Cas-analyzer	Web service	Web GUI	Park et al. [6]
CRISPRMatch	Linux	Command line	You et al. [23]
CRISPResso2	Linux, Docker	Command line	Clement et al. [5]
CRIS.py	Linux, Windows, macOS	Command line	Connelly et al. [8]

To provide a more convenient tool for novice users, we developed CRISPR-GRANT, a stand-alone graphical CRISPR indel analysis tool with easy installation and cross-platform support, including Linux, Windows, and macOS. CRISPR-GRANT provided a straightforward GUI to guide the analysis of single/pooled amplicons and whole-genome sequencing (WGS) by simple click-and-run. Moreover, the program also exhibited highly efficient run-time compared with representative benchmark tools currently available. Together, CRISPR-GRANT would be a valuable addition to the current toolkits that significantly lower the barrier for wet-lab researchers to conduct indel analysis from large NGS datasets.

## Implementation

### Graphical user interface

CRISPR-GRANT used ui library (version 0.9.4) to make cross-platform GUI, ggplotnim (version 0.3.18) for figure plotting and other Nim libraries. The icon of CRISPR-GRANT was derived from the online website flaticon (flaticon.com). Executable binary files for each operating system (OS) were compiled from source codes on the corresponding OS.

### Pre-processing

Fastp [9] was first used for quality control of the input RAW FASTQ files and low-quality reads will be removed. The quality score of nucleic bases could either be indicated by users or kept default. The QC reports were saved to HTML and JSON format. For pair-ended amplicon(s) sequencing data, the reads will be merged by FLASH [10] and saved as a compressed FASTQ file. The resulting merged reads or the qualified single-ended reads will then be mapped to the reference by BWA-MEM [11] and the resulting SAM file will be sorted to BAM by samtools [12]. VarScan2 will then analyze the BAM file for consensus and variants [13].

### Quantification and visualization

The quantification and visualization parts are mainly written in Nim with the ggplotnim library (https://github.com/Vindaar/ggplotnim). The resulting SAM file from BWA-MEM was used for the quantification of different indel types. The counting result was sorted by the number of reads of each indel type and saved as a table file with the “.csv” extension. Then the modified and un-modified reads were calculated using a program written in Nim and saved as a table file with the “.csv” extension. Indels with the top most number of reads (termed top n reads) were conducting multiple sequence alignment using mafft [14]. The resulting alignments were saved as a FASTA file with the “.fasta” extension and then plotted with a custom program written in Nim.

### Data simulation and performance comparison

Simulated data was generated by ART [15]. Performance comparison tests were processed on simulated data using a desktop computer (Ubuntu 16.04, Intel Core i7-8700K CPU 3.7 GHz and 64 GB of RAM).

**Fig. 1:**
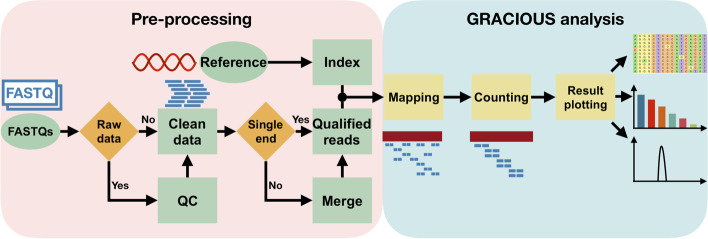
CRISPR-GRANT enables indel analysis from raw sequencing data to result figures. Schematic showing the main pipelines used in CRISPR-GRANT: FASTQ files pre-processing, mapping to reference genome, reads count, visualization of alignment and indel distribution, etc.

The working flow diagram of CRISPR-GRANT is shown in Fig. 1.

## Results

### Graphical user interface on multiple platforms

**Fig. 2:**
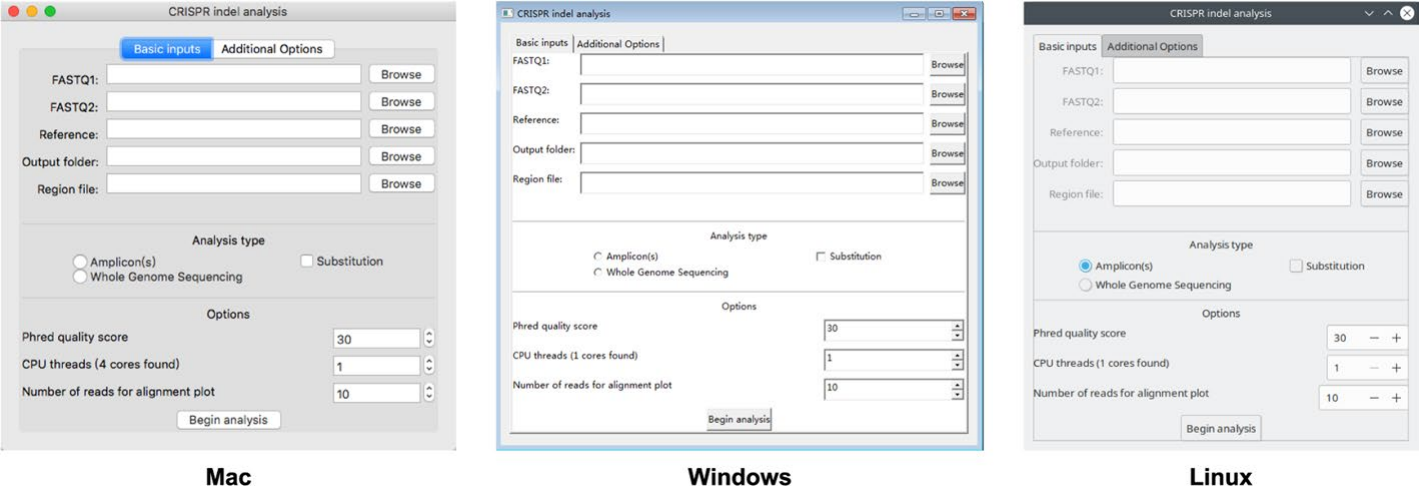
CRISPR-GRANT provides intuitive GUI for CRISPR indel analysis on multiple platforms. CRISPR-GRANT could be installed and run on MacOS, Windows, and Linux. Screenshots showing the GUI running on three main desktop operating systems: Mac, macOS (10.13); Windows, Windows 7 (sp1); Linux, GNU/Linux (openSUSE)

CRISPR-GRANT was written to provide a straightforward cross-platform GUI, where the users could complete indel analysis by simple point-and-click (Fig. 2). The GUI consists of two parts: one is for basic inputs where the FASTQ file(s), reference sequence file, and output folder could all be input by simple mouse-clicking. Default options are given through GUI and support custom changes by users. The other mode is for advanced users with additional options. For users who would like to set more parameters for the tools within the analysis pipeline, additional options would pass to those tools directly. A universal GUI was provided for single-end or paired-end sequencing data from amplicon, pooled amplicons, and allele-specific analysis, supporting analysis for genome editing events from Cas9/Cpf1 and base-editors. The overall procedure was the same for all the analyses. All the needed data could be given through the same GUI with similar operations and easy to learn and use. The only required inputs are: (1) FASTQ file(s); (2) reference sequence(s); (3) the output folder for analysis results. For single-end sequencing, the respective FASTQ file is required. As for paired-end sequencing results, two separate FASTQ files, one for each end, should be provided. The input reference sequence is needed in FASTA format. Depending on the intended analysis, one (amplicon), two (allele-specific, e.g.), or more (pooled amplicons) sequences could be put into a single reference file. The overall procedure is kept the same for all the different analyses, for common users to run the processing with minimum guidance.

In addition, CRISPR-GRANT also provides command-line tools for more advanced users. All the tools in the pipeline are independent and could be used separately when the correct input data are given, which is developed in case of special requirements from end-users.

### Data processing and visualization

**Fig. 3:**
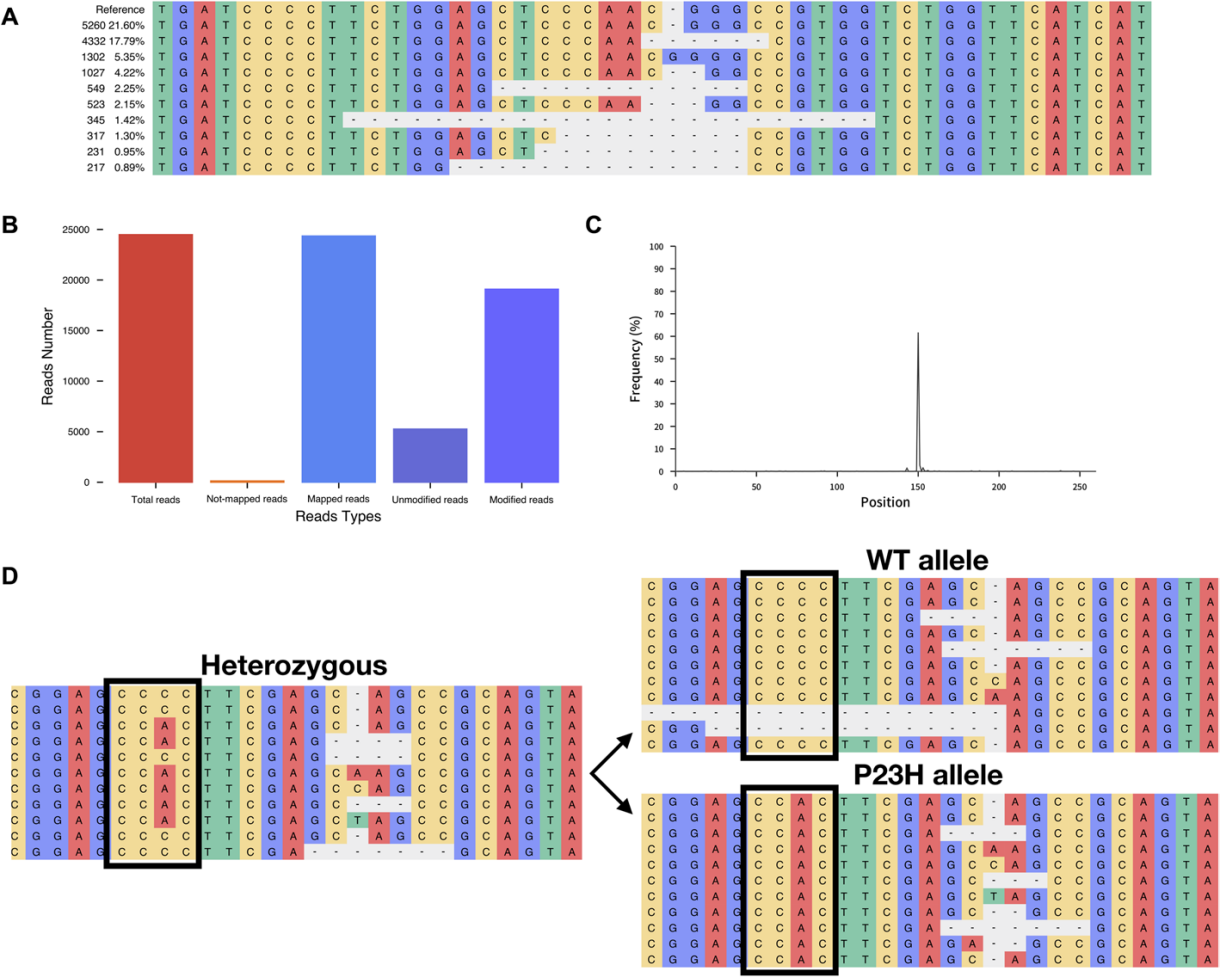
Features for CRISPR-GRANT in indel analysis. **A** Alignment and quantification plot of reads against the reference sequence. Reference was placed as the first sequence and all the other detected reads were aligned with labeled percentile quantification. **B** Summary bar plot showing the number of different reads processed. Total reads included reads not-mapped and mapped to reference. Reads mapped to reference consisted of modified and un-modified reads. **C** Frequency distribution of indels along the reference sequence. **D** Heterozygous alleles (left) could be assigned to each allele using CRISPR-GRANT for quantifying multiple alleles of a given genomic locus. Sample data: sequencing results from SaCas9-KKH editing of the *Rho* gene in P23H heterozygous mice [19]

To ensure convenience for novice users, CRISPR-GRANT is designed to be directly applied to raw sequencing data generated from NGS, with pre-processing carried out by CRISPR-GRANT’s pipeline. The final mapping results will be quantified, and the corresponding high-resolution plots will be produced. For the plots, sample visualizations are provided, such as top n reads (the number n was customized) alignment with reference (Fig. 3A), distribution of reads counts (total reads, mapped reads, modified and un-modified reads) (Fig. 3B), frequency of indels at each position along with the reference (Fig. 3C) and allele-specific analysis (Fig. 3D).

### Whole-genome sequencing analysis from raw data

Since genome-wide identification of CRISPR editing events (such as off-target effects) has become a common strategy for evaluating on-target and off-target efficiency [16], CRISPR-GRANT was also designed to support one-step analysis from raw WGS data, a unique feature compared with other available indel analysis tools. CRISPR might cause mutations beyond expectation among the whole genome. Pooled amplicon sequencing could evaluate mutations at some specific sites on the genome, such as prediction tools or other assays, while most of the genome remained no investigation. Though CRISPResso or CRISPResso2, for example, had provided utilities, CRISPRessoWGS, analyzing genome editing from WGS data, however, BAM file(s) aligned to genome reference still must be provided, which expected users to have bio-informatics background. Therefore, none of those CRISPR analysis tools currently, as far as we know, could analyze indel mutations of whole-genome for wet-lab researchers from raw WGS data. Using CRISPR-GRANT, the users only need to provide raw sequencing data (single-end or paired-end FASTQ files), reference genome sequence and a file containing regions of interest to analyze through point and click. This function was especially useful in investigating and quantifying CRISPR indel frequencies of potential target or off-target sites on the whole genome for biologists. This feature enables the user to systematically identify and evaluate DNA mutations caused by CRISPR/Cas system, supporting both regular Cas9/Cpf1 and base-editors. When comparing with one of the benchmark programs that have been widely used, CRISPResso2 for example, pre-processing of WGS data, such as alignment to the reference genome, is expected for CRISPResso2 before analysis, which would require the users to have some bioinformatics background and finish the procedure using command line when running the program. In CRISPR-GRANT, however, the end-users would only need to provide raw sequencing data (single-end or paired-end FASTQ files), the reference genome sequence, and a file containing regions of interests to analyze, through the simple point-and-click, which would be especially useful for lab scientists to analyze and quantify indel frequencies of potential off-target sites on the genome-scale.

### Performance comparison with representative benchmark tools.

**Fig. 4:**
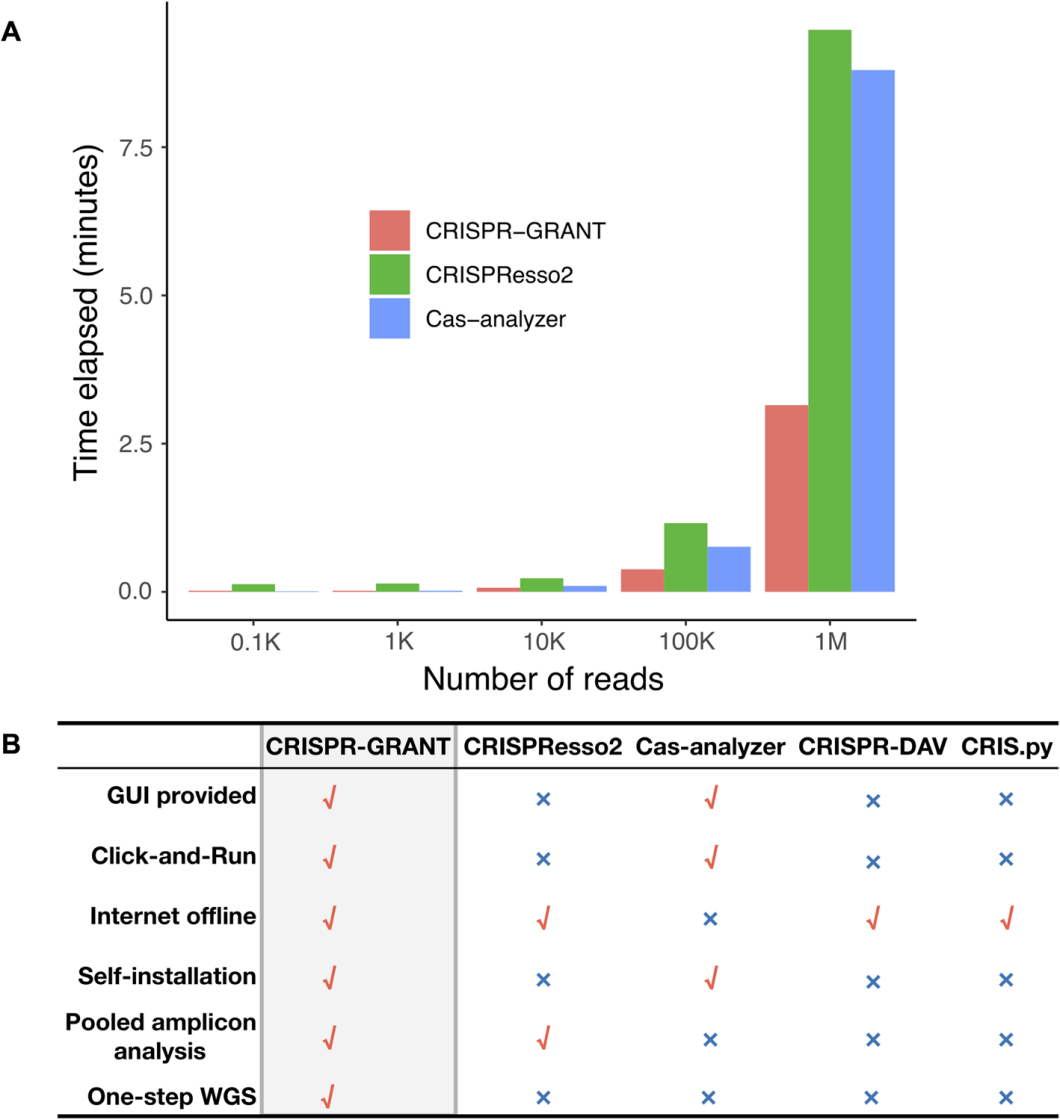
CRISPR-GRANT is an efficient stand-alone program for large-scale indel data processing. **A** CRISPR-GRANT outperformed other representative tools currently available in processing different scales of data. All tests were taken using default parameters. K: kilo; M: mega. Tests were done on simulated data generated by ART using Ubuntu 16.04 with i7-8700K (3.7 GHz) and 64 GB RAM. **B** Comparison of CRISPR-GRANT with other published CRISPR indel analysis tools. CRISPR-GRANT is an efficient and versatile tool for novice laboratory scientists

To evaluate the performance of CRISPR-GRANT on data analysis, a cross-comparison of single amplicons analysis was performed with representative benchmark tools, CRISPResso2 and Cas-analyzer, using the same sample data [4]. When analyzing small or medium-size data, all the tools could finish the run within a reasonable time frame, while CRISPR-GRANT cost the least time to complete the analysis. When analyzing simulated data containing a large number of reads (1 M (million) reads), CRISPR-GRANT was the most efficient and only took about half an hour to complete the analysis. (Fig. 4A).

## Conclusions

In this study, we developed a stand-alone indel analysis tool, CRISPR-GRANT, for the efficient evaluation of CRISPR/Cas-mediated genome editing. CRISPR-GRANT supports easy installation on multiple platforms, including macOS, Windows, and Linux, and provides a user-friendly GUI to guide the analysis process for common novice lab researchers. Moreover, the program offers one-step analysis for whole-genome sequencing data by simple click-and-run and exhibited more efficient data processing speed when compared with other available benchmark programs. Therefore, CRISPR-GRANT may serve as a valuable addition to the current toolkits for CRISPR/Cas-mediated genome editing analysis.

With the application of next-generation sequencing technology, especially the recent addition of single-cell sequencing [17] and spatial profiling, the research broadness and data scale which traditional biology scientists handle in the lab have been vastly expanded. Therefore, it is now more important than ever for researchers to rely on efficient bioinformatics programs and pipelines to process and analyze NGS datasets. This is also the case for CRISPR/Cas-mediated genome editing, where the potential impact of off-target effects receives increasing attention, and evaluation of mutations on the genome-wide scale has become a common practice in the lab. Most of the programs currently available use the command-line and Linux-system-based approach to run the analysis, which usually requires the researchers to have certain bioinformatics training and thus is inconvenient for junior students and regular scientists. With this in mind, CRISPR-GRANT was designed to meet the need for large-scale data analysis for novice researchers. Especially for whole-genome sequencing data, CRISPR-GRANT offers one-step WGS analysis from raw data and run with clear GUI guidance, a unique feature compared with other tools. Other features of CRISPR-GRANT include easy installation on multiple systems, off-line use without the need of uploading sensitive data, and high running efficiency (Fig. 4B). Moreover, the program is also compatible with other genome editing technologies such as TALENs and ZFNs [18]. Therefore, CRISPR-GRANT is a user-friendly and GUI-enabled program that would be very helpful for lab researchers to analyze both on-target and off-target genome editing results.

Meanwhile, it is also worth noting that a few improvements for CRISPR-GRANT would warrant further exploration. One of such is to expand the analytical capabilities for WGS data. The current version of the program requires the end-users to provide specific target sites for alignment visualization, while ideally, it would be more exciting if the program could plot alignment genome-wide. Secondly, improvement is also warranted on the analysis accuracy by filtering the PCR artifacts from amplified single or pooled amplicons. As PCR is used for the amplification process, which would occasionally bring artificial mutations into the amplicon, a filtering process will help to remove the potential noise from indel analysis. Besides, the options for output figures could also be a useful addition to the current toolkit.

In summary, CRISPR-GRANT is a stand-alone and versatile tool that provides efficient indel analysis capability for both single, pooled amplicons and WGS datasets, supporting a variety of CRISPR/Cas systems as well as other genome editing technologies. With its user-friendly GUI feature, CRISPR-GRANT will be a valuable tool to meet the need of novice experimental biologists for analyzing small- or large-scale genome edits.

### Availability and requirements

Project name: CRISPR-GRANT. Project home page: https://github.com/fuhuancheng/CRISPR-GRANT. Operating system: Windows, mac OS and Linux. Programming language: Nim. License: GNU GPL v3. Any restrictions to use by non-academics: Terms stated in GNU GPL v3.

## Data Availability

CRISPR-GRANT binaries are freely available as Supplementary Software and free to download at https://github.com/fuhuancheng/CRISPR-GRANT/releases for Linux, macOS and Windows. CRISPR-GRANT source code is licensed under the GPLv3 license and free to download and use at https://github.com/fuhuancheng/CRISPR-GRANT. Pooled amplicon data analysed during the current study are available in the NCBI Short Read Archive (SRP109554).

## Abbreviations

CRISPR: Clustered regularly interspaced short palindromic repeats
NHEJ: Non-homologous end joining
HDR: Homology-directed repair
NGS: Next-generation sequencing
GUI: Graphic user interface
QC: Quality control
WGS: Whole genome sequencing
NA: Not available
